# Optimization of conditions for apnea testing in a hypoxemic brain dead patient

**DOI:** 10.5935/0103-507X.20190015

**Published:** 2019

**Authors:** Bárbara Vieira Carneiro, Guilherme Henrique Garcia, Larissa Padrão Isensee, Bruno Adler Maccagnan Pinheiro Besen

**Affiliations:** 1 Unidade de Terapia Intensiva Clínica, Disciplina de Emergências Clínicas, Departamento de Clínica Médica, Hospital das Clínicas, Faculdade de Medicina, Universidade de São Paulo - São Paulo (SP), Brasil.; 2 Unidade de Terapia Intensiva Clínica Médica, Divisão de Fisioterapia, Hospital das Clínicas, Faculdade de Medicina, Universidade de São Paulo - São Paulo (SP), Brasil.

**Keywords:** Brain death, Critical care, Hypoxia, Prone position, Respiration, artificial, Tissue donors

## Abstract

We report the case of a patient in whom brain death was suspected and associated with atelectasis and moderate to severe hypoxemia even though the patient was subjected to protective ventilation, a closed tracheal suction system, positive end-expiratory pressure, and recruitment maneuvers. Faced with the failure to obtain an adequate partial pressure of oxygen for the apnea test, we elected to place the patient in a prone position, use higher positive end-expiratory pressure, perform a new recruitment maneuver, and ventilate with a higher tidal volume (8mL/kg) without exceeding the plateau pressure of 30cmH_2_O. The apnea test was performed with the patient in a prone position, with continuous positive airway pressure coupled with a T-piece. The delay in diagnosis was 10 hours, and organ donation was not possible due to circulatory arrest. This report demonstrates the difficulties in obtaining higher levels of the partial pressure of oxygen for the apnea test. The delays in the diagnosis of brain death and in the organ donation process are discussed, as well as potential strategies to optimize the partial pressure of oxygen to perform the apnea test according to the current recommendations.

## INTRODUCTION

"Brain death" is the terminology used to express the condition of irreversible coma associated with the absence of body reflexes and the occurrence of persistent apnea.^(^^1^^)^ Diagnosis of brain death and management of the potential donor are common in intensive care. Establishing a diagnosis of brain death is a complex process and must be performed with precision. It is a condition that is not well understood by relatives and nonspecialists, and it involves medical, ethical, and legal precepts.^(^^2^^)^

Worldwide, the stages that define brain death are not uniform, and cultural and legal differences may even exist within a single country. In Brazil, the Federal Council of Medicine Resolution 2,137/2017 determines the methodology for the diagnosis of brain death.^(^^3^^)^

This new Brazilian resolution requires that a single apnea test be administered by one of the physicians responsible for the clinical examination. The resolution also requires that patients be ventilated with an inspired oxygen fraction (FiO_2_) of 100% for at least 10 minutes and that mechanical ventilation be optimized to reach a partial pressure of oxygen (PaO_2_) ≥ 200mmHg and a partial pressure of carbon dioxide (PaCO_2_) between 35 and 45mmHg.^(^^3^^)^

Here, we report the case of a patient who was suspected to have brain death associated with atelectasis and moderate to severe hypoxemia. The goal of this report is to demonstrate the difficulties in obtaining safe levels of PaO_2_ for the apnea test, as well as potential hypoxemia management strategies that can be used to optimize oxygenation in this context.

## CASE DESCRIPTION

A 57-year-old male, who was previously hypertensive and diabetic, was treated with hydrochlorothiazide, enalapril, and metformin as an outpatient and was admitted to the Hospital das Clínicas da Faculdade de Medicina da Universidade de São Paulo with dysarthria, left hemispatial neglect, and complete and proportional left hemiplegia. After formulating the hypothesis of stroke, the stroke protocol was activated. Computed tomography (CT) of the skull showed no signs of bleeding, and computed tomography angiography of the intra and extracranial arteries revealed occlusion at the origin of the right middle cerebral artery with caudal extension to the ipsilateral internal carotid artery.

**Figure 1:**
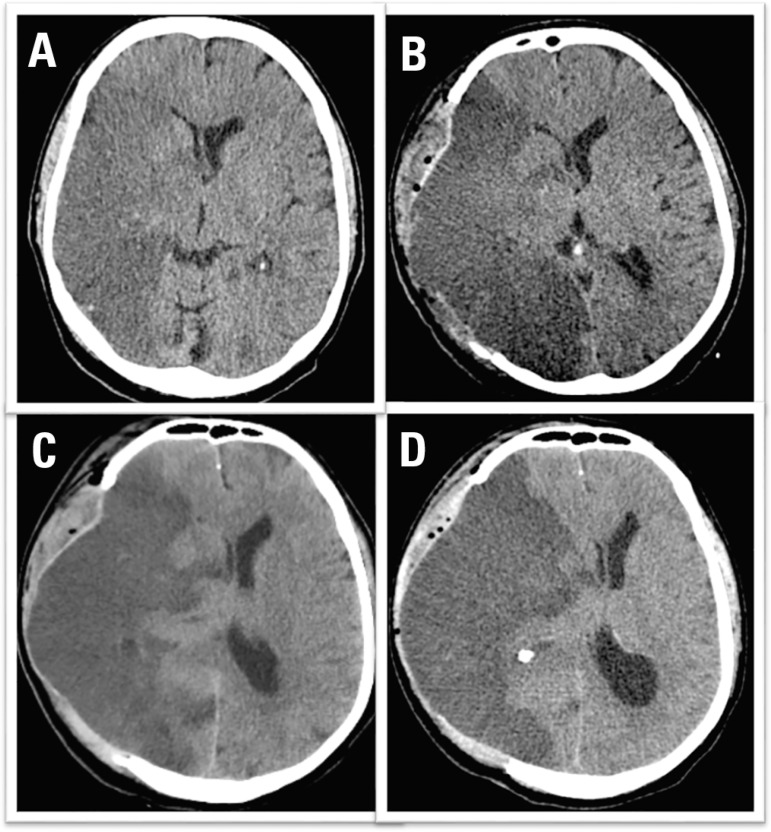
Head CT scans before and after decompressive craniectomy (sections at the level of the septum pellucidum). (A) Computed tomography of the skull with 18 hours of evolution. (B) Postoperative computed tomography scan (36 hours of evolution) revealed good surgical results. (C) Computed tomography of the skull with 60 hours of evolution and a moderate midline deviation. (D) Computed tomography of the skull with 96 hours of evolution and a significant midline deviation.

The patient underwent thrombolysis after 3 hours and 53 minutes. During the observation period in the emergency department, the level of consciousness of the patient decreased, and thus he required endotracheal intubation. The control CT scan of the skull showed right hemispheric edema, which was consistent with malignant middle cerebral artery infarction (Figure 1). A right fronto-temporo-parietal decompressive craniectomy with classic durotomy was indicated and performed within 24 hours of the stroke, and the patient was transported to the intensive care unit (ICU) after the surgical procedure.

Despite the extensive craniectomy, neurological deterioration increased over the next several days. Since the patient underwent the most effective therapy to control intracranial hypertension without success, we chose not to implement other measures for intracranial hypertension. On the fourth day of his ICU stay, the patient lost all body reflexes, was hypotensive and was likely brain dead. The tomographic series is described in figure 1. Then, at approximately 8 o'clock, we initiated life-support measures and the brain death protocol for this potential donor and notified the organ and tissue procurement service of the hospital.

### The problem

The patient became hemodynamically unstable during the hours following brain death. We performed volume expansion and initiated an infusion of noradrenaline and vasopressin. The bedside echocardiogram did not show significant changes in left or right ventricular function. We decided to start hormonal resuscitation with the enteral administration of thyroid hormone (levothyroxine 100µg), hydrocortisone (50mg every 6 hours), and infusion of glucose and insulin (0.5U/kg/hour). The patient achieved hemodynamic stability but with a moderate dose of vasopressors.

**Figure 2:**
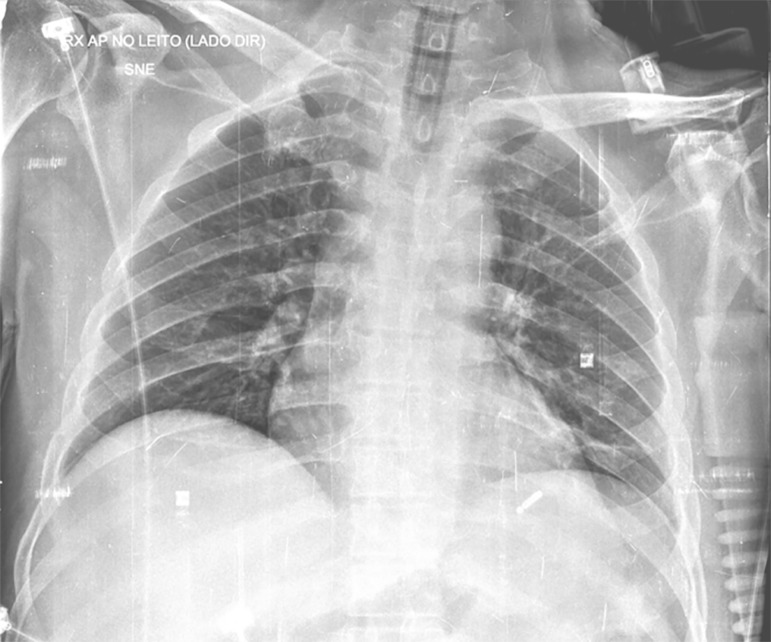
Anteroposterior chest X-ray obtained while the patient was in bed.

Despite hemodynamic stabilization, the patient also presented with moderate hypoxemia (PaO_2_/FiO_2_ ~ 110), and thus it was difficult to perform the apnea test. No clinical evidence of respiratory infection was observed. An ultrasound showed pulmonary collapse in both lung bases, which was not observed in the chest X-ray obtained the previous day (Figure 2).

### The solution

We maintained the patient in volume-controlled mode with a respiratory rate (RR) of 22 bpm (breaths per minute), a positive end-expiratory pressure (PEEP) of 5cmH_2_O, an FiO_2_ of 1, a tidal volume (TV) of 370mL, and an initial PaO_2_ of 109mmHg. We used a closed suction system and attempted to perform a recruitment maneuver with PEEP elevation up to 20cmH_2_O, which was interrupted due to hemodynamic instability and worsening of oxygenation. At that time, we maintained the final PEEP at 8cmH_2_O.

To achieve safer levels of PaO_2_, we placed the patient in a prone position. A PaO_2_/FiO_2_ ratio of 171mmHg was obtained once the patient was in a prone position along with an RR of 22bpm, a PEEP of 8cmH_2_O, an FiO_2_ of 0.4, and a TV of 370mL.

Since we did not reach the values stipulated by the new resolution, while keeping the patient prone, we chose to use a higher FEEP (15cmH_2_O) associated with the recruitment maneuver and increased the FiO_2_ to 1, which maintained the RR at 22bpm and the TV at 370mL. This way, we obtained a PaO_2_ of 165mmHg.

In the last attempt, we chose to increase the TV up to the plateau pressure limit (8mL/kg for maximum plateau of 30cmH_2_O), with TV of 480mL, while not modifying the other parameters. These measures resulted in PaO_2_/FiO_2_ of 241mmHg and PaCO_2_ of 41mmHg, thus we started the apnea test.

Table 1 summarizes the ventilatory parameters and the gasometric findings in each stage.

**Table 1:** Blood gas and evolution of respiratory mechanics for initiation of the apnea test

Variable	Protective VM	Protective MV + Prone	Protective MV + Prone + high PEEP	Protective MV + Prone + high PEEP + TV 8mL/kg	Posttest
Time	10:38 a.m.	1:59 p.m.	3:24 a.m.	4:21 p.m.	5:39 p.m.
Blood gas parameters					
pH	7.3	7.29	7.24	7.26	6,97
PaO_2_	109	68.4	165	241	243
PaCO_2_	40.7	39.3	42.2	41.3	93.7
Bicarbonate	19.5	18.5	17.8	17.9	20.6
SBE	- 6	- 6.8	- 8.4	- 8.2	- 14.2
SatO_2_	97.2	93.9	99.1	85.8	98.5
Ventilatory parameters					
PEEP	5	8	15	15	10
FiO_2_	1	0.4	1	1	1
PaO_2_/FiO_2_ ratio	109	171	165	241	-
Respiratory rate	22	22	22	22	-
TV	370	370	370	480	-
Plateau pressure	20	20	25	30	-
ΔP	15	12	10	15	-

MV - mechanical ventilation; PEEP - positive end-expiratory pressure; TV - tidal volume; PaO_2_ - partial pressure of oxygen; PaCO_2_ - partial pressure of carbon dioxide; SBE - standard base excess; SatO_2_ - oxygen saturation; FiO_2_ - fraction of inspired oxygen; ΔP - lung-distending pressure.

### The apnea test

The apnea test is based on the absence of respiratory movements after maximal stimulation of the respiratory center by hypercapnia (PaCO_2_ > 55mmHg).^(^^1^^)^ The test should be stopped whenever the following is observed: (1) respiratory movements (negative apnea test), (2) hemodynamic instability, or (3) severe hypoxemia.^(^^1,3,4^^)^

Since the patient required several maneuvers to achieve adequate oxygenation before the test, we elected to perform the test with the patient in a prone position with a continuous positive airway pressure (CPAP) valve placed in the T tube through which oxygen flowed at a rate of 12L/minute, as previously described.^(^^5^^)^ From a hemodynamic standpoint, the patient tolerated the test, as he maintained 100% saturation throughout the test; moreover, posttest arterial blood gas analysis confirmed the validity of the test (Table 1).

### Evolution

We completed the apnea test and the first clinical trial at 5:30 p.m. on the same day, which resulted in a 10-hour delay in relation to the suspected diagnosis of brain death. The second clinical trial was initiated at 7:15 p.m. by another specially trained intensivist. Complementary examination (transcranial Doppler) showed total cerebral circulatory arrest. The patient's family members agreed that the patient could donate his organs, and procurement was scheduled for the following morning. However, overnight, the patient developed circulatory arrest due to refractory shock, and no organ procurement was possible.

## DISCUSSION

This case demonstrates the difficulty imposed by hypoxemia, a common condition in neurocritical patients, in the determination of brain death. The need to optimize the PaO_2_ before performing the apnea test delayed the diagnosis by 10 hours and contributed to the loss of potential donated organs due to circulatory arrest. Despite this outcome, we described a set of additional maneuvers to optimize the pretest PaO_2_ to avoid missing the diagnosis of brain death. Finally, we described, for the first time, the performance of the apnea test with the patient in a prone position to maintain adequate oxygenation during the procedure.

The apnea test is essential in the process of determining brain death. However, this test poses a risk to patients due to the possibility of hypotension, hypoxemia, arrhythmias, and cardiac arrest. The occurrence of any of these conditions can lead to termination of the test and delayed diagnosis of brain death. North American data show that 7% of patients with suspected brain death are unable to initiate the apnea test due to hemodynamic instability or hypoxemia, while the test is terminated in 3% for the same reasons.^(^^6^^)^

Some prerequisites have been established by the new Brazilian guidelines for initiation of the apnea test: body temperature > 35ºC, systolic blood pressure ≥ 100mmHg or mean arterial pressure ≥ 65mmHg, PaCO_2_ between 35 and 45mmHg, and PaO_2_ ≥ 200mmHg.^(^^3^^)^ However, no consensus has been established on what the required levels of PaO_2_ should be for performing the apnea test. Wijdicks et al. (2008) did not observe a significant difference between the levels of PaO_2_ among those who completed or the apnea test and those who did not.^(^^6^^)^ From a clinical point of view, although a minimal PaO_2_ is useful to avoid hypoxemia during the test, the cutoff value will not always be reached, and there are other more effective ways to maintain oxygenation such as performing the CPAP test.^(^^5^^)^ Despite this, the current resolution requires that the apnea test be preceded by attempts to optimize oxygenation to increase the safety of the test. In contrast, in other countries, a complementary examination is mandatory only for patients who do not tolerate the apnea test or when the results of the neurological exam are questioned. In Brazil, the complementary exam is mandatory for all cases, and healthcare personnel do not have the option to not perform the apnea test, which can result in the loss of potential donors.

The respiratory support recommended for a potential donor involves the following set of actions: (1) a TV of 6 - 8mL/kg of the patient's predicted weight without exceeding the plateau pressure of 30cmH_2_O, (2) a PEEP of 8 - 10cmH_2_O, (3) a closed suction system, (4) CPAP apnea test, and (5) recruitment maneuvers in case of ventilator disconnection. This set of actions was evaluated in a randomized clinical trial, which demonstrated an absolute increase of 27% in the number of lungs procured for transplant^(^^7^^)^ and is recommended by the Brazilian guidelines for maintaining potential donors.^(^^8^^)^

This strategy was not sufficient to optimize oxygenation to safe levels in the case presented. Thus, we performed other measures that can lead to better oxygenation. We started with the patient in the prone position.^(^^9^^)^ After 7 hours of mechanical ventilation in the prone position, two alveolar recruitment maneuvers, maintenance of high PEEP,^(^^10^^)^ and an increase in the TV of 8mL/kg, we obtained an increase in PaO_2_ compatible with the CPAP apnea test, which was performed with the patient in the prone position. This set of maneuvers allowed for a safer apnea test with adequate levels of PaO_2_ before and after the test.

## CONCLUSION

The present report aimed to highlight the problem of hypoxemia in neurocritical patients with suspected brain death and the implications on the performance of the apnea test. We demonstrated a variety of bedside strategies that can be used so that the apnea test can be performed safely and effectively, which may reduce the incidence of missed diagnosis of brain death due to the difficulty in performing the test as recommended in the resolution of the Brazilian Federal Council of Medicine.
